# Ethical guidelines for Sami research: the issue that disappeared from the Norwegian Sami Parliament's agenda?

**DOI:** 10.3402/ijch.v74.27024

**Published:** 2015-04-08

**Authors:** Vigdis Stordahl, Grete Tørres, Snefrid Møllersen, Inger-Marit Eira-Åhren

**Affiliations:** 1 Sámi Norwegian National Advisory Unit on Mental Health and Substance Use, Finnmark Hospital Trust, Hammerfest, Norway; 2 Norwegian Reindeer Herders’ Organization, Tromsø, Norway

**Keywords:** research ethics, indigenous, Sami, Norway

## Abstract

**Background:**

In recent decades many indigenous communities, policy makers and researchers worldwide have criticized the academic community for not being aware of the specific challenges these communities have faced and still are facing with regard to research. One result of the decades of discourse in indigenous communities is the development in many Western countries of indigenously sensitive ethical research guidelines. In 1997 the Sami Parliament (SP) in Norway reached a unanimous decision that ethical guidelines for Sami research had to be drawn up. Such guidelines are however still to be created.

**Objectives:**

The objectives of this article are to enquire into what happened to the Norwegian SP's decision of 1997 and to reflect on why the issue seems to have disappeared from the SP's agenda. Finally, we consider whether research ethics is to be a subject for the research community only.

**Methods:**

A review of parliamentary white papers on research and SP documents relating to research ethics.

**Findings:**

The response to the SP's decision in 1997 took place in two different channels, both of them national, namely the research ethics channel and the political channel. Thus, there were actually two parallel processes taking place. In spite of nearly two decades of reports, the concept of the participation of indigenous communities in research is still not an integral part of Norwegian ethical guidelines.

**Conclusions:**

The issue of indigenously sensitive research ethics seems to have disappeared from the SP's agenda and the research ethics review system with regard to Sami research is with minor adjustments the same as when the SP asked for a revision.

In recent decades many indigenous communities, policy makers and researchers worldwide have criticized the academic community for not being aware of the specific challenges these communities have faced and still are facing with regard to research. This also holds true for the Nordic countries where the Sami population has been seen as an interesting research topic for at least two centuries (1). One result of the decades of discourse in indigenous communities is the development in many Western countries of indigenously sensitive ethical research guidelines (2–7).

In 1997 research was on the agenda of the Sami Parliament (SP)^1^ in Norway. With regard to research ethics, the outcome was a unanimous decision that ethical guidelines for Sami research had to be drawn up and that a separate Sami research ethics committee needed to be established.

One of the arguments of the SP was that research ethics were inseparable from the social and cultural context of the people researched. The SP also argued that the national ethics committees did not have adequate competence and insight to deal adequately with the question of research ethics involving Sami society (8). With this statement, the Norwegian SP argued in line with the international indigenous discourse on research ethics of the time. The SPs in Finland and Sweden have not made any decision as to research ethics, but both bodies have in the autumn of 2014 discussed the issue.^2^

Since 1990, Norway has had three National Committees for Research Ethics that together cover all disciplines: one for Research Ethics in the Social Sciences and the Humanities, one for Research Ethics in Science and Technology and one for Medical and Health Research Ethics (NEM) (9). NEM is an advisory and appeals body for the seven Regional Committees for Medical and Health Research Ethics. The regional committees evaluate all medical and health research projects, while NEM gives its opinion on issues that are more a matter of principle.

The Norwegian National Committees for Research Ethics have developed both general and subject-specific ethical guidelines. Indigenous peoples are however not specifically mentioned in any of these guidelines, not even in the newly published general ethical guidelines.

Almost two decades have passed since the SP decision, but still there are no specific ethical guidelines for research into Sami society. Since the SP in 1997 argued that the national ethics committees did not have adequate competence and insight to evaluate satisfactorily questions of research ethics involving the research interests of Sami society and these committees are still responsible for reviewing such research projects, we became interested in finding out what actually happened to the SP decision of 1997.

We thus enquired at the SP administration for documents relating to research ethics and searched in the Norwegian Government's white papers (Stortingsmelding) on research with the key words “research ethics,” “Sami research ethics,” “Sami research.”

In this article we will present the findings of our review of these documents followed by some reflections on why the issue seems to have disappeared from the SP's agenda. Inspired by the international indigenous discourse on research ethics as well as our own experiences in developing a research project in collaboration with the Sami reindeer herding community (10), we conclude with some reflections as to whether research ethics is to be a subject for the research community only.

When indigenously sensitive research ethics are on the agenda in international research fora, we have noted some surprise that Norway focuses so little on this. We therefore entertain a hope that this article will shed light on the reasons for this lack of focus, and will also encourage the research community to see their responsibility in getting the issue back on the agenda again.

## Two parallel processes

**Fig. 1:**
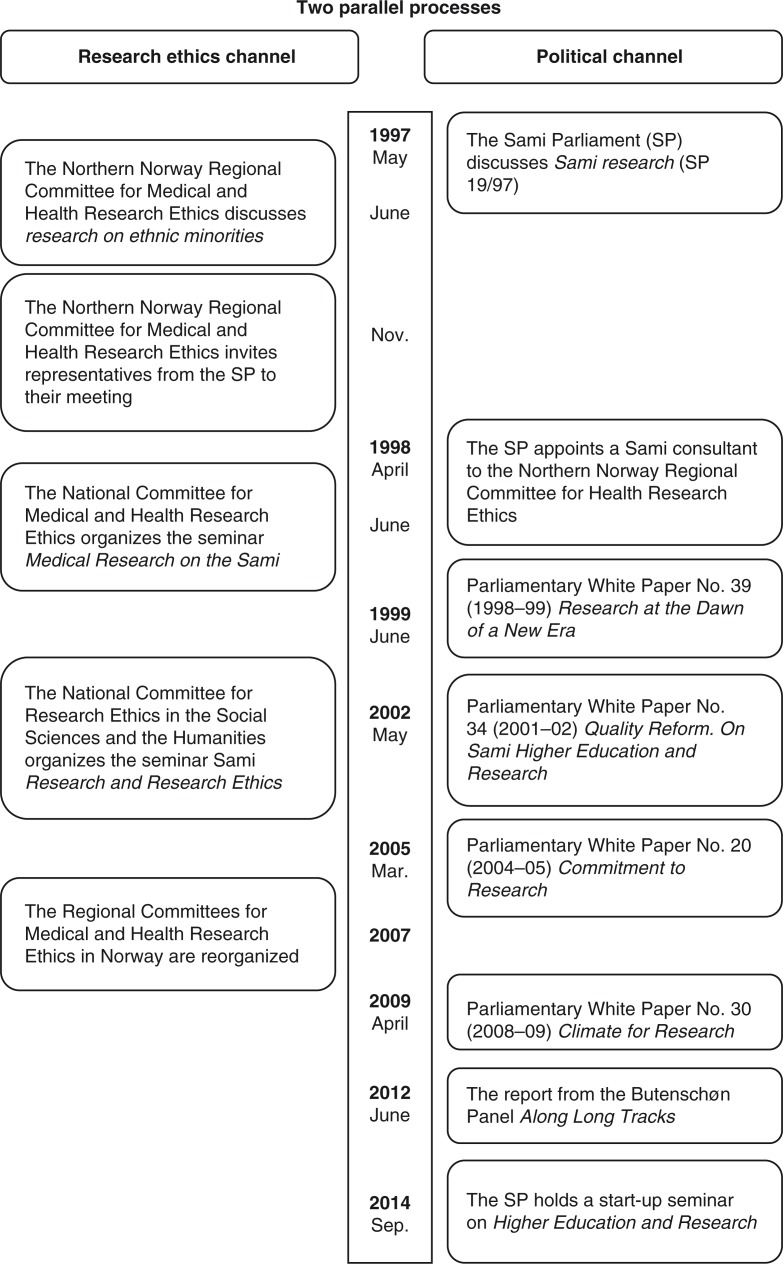
Two parallel processes.

In tracking down the case, it became evident that the response to the SP's decision in 1997 took place in two different channels, both of them national. One channel was the research ethics channel, while the other was the political channel. Thus, there were actually two parallel processes taking place (Fig. 1).

## The research ethics channel

The Northern Norway Regional Committee for Medical and Health Research Ethics (REC V) responded immediately to the SP's decision. In June 1997 research on ethnic minorities was on their agenda and they invited the head of administration of the SP to open the discussion. Later the same year they held their meeting in the location where the SP has its main office and invited representatives of the SP to meet with them. REC V stated that they found it important to avoid any conflict situations between them and the SP. They asked to be contacted when the panel on a separate Sami research committee was to be set up. They would also like to consult the SP when epidemiological research programs were planned, they approved of the use of a Sami consultant in evaluating smaller projects and they would propose to the National Committee for Medical and Health Research that they be the committee responsible for evaluating all biomedical health research projects in Norway involving Samis.

The National Committee for Medical and Health Research Ethics also responded and in June 1998 they arranged a seminar at the University of Tromsø on ethics in Sami research. In September the same year, they invited several institutions to discuss how they best could follow up this issue.

The outcome of this for health research was that from 1998 to 2007 the REC V had national responsibility to give ethical evaluations of all biomedical research involving Samis. The committee could ask the advice of a Sami consultant appointed by the SP. When the National Committees for Medical and Health Research Ethics were reorganized in 2007, these arrangements were discontinued. All research applications were now to be reviewed by the regional REC the applicant belonged to and none of the RECs was to have a specific responsibility for Sami health research.

The National Ethics Board for the Humanities and Social Sciences also responded and in May 2002 they held a seminar on Sami research and research ethics. A report with the papers presented at the seminar was later published (11). In 2005 they issued revised ethical guidelines. Chapter C focuses on the responsibility researchers have towards groups and institutions, but Sami or indigenous people are not specifically mentioned here.

## The political channel

The SP sent their decision to the ministry responsible for research asking them to ensure that the issue was followed up and coordinated with the ongoing work on a report by the Research Council of Norway (RCN). Two years later, in June 1999, we find the response of the Government. In their white paper Research at the Dawn of a New Era (12) to the Parliament, they state that they do not see the need for establishing separate bodies, be it a Nordic Sami research council or an ethics committee. In their opinion it was natural to use the opportunities found in the national research system. However, the ministry would consider appointing members with what they called Sami knowledge to the national committees of research ethics. The RCN was asked to propose how teaching in Sami subjects could be organized on a Nordic basis.

The next time we see Sami research mentioned in a white paper on research is in March 2002 (13). The Government refers to the fact that the RCN now has a program on Sami research and that research ethics is to be given priority. There is however no mention of the SP decision from 1997.

When a new white paper on research, Commitment to Research (14), was launched in March 2005, the RCN were asked to take the initiative to establish a Nordic panel with the mandate to present a report on a Nordic Sami research council. The issue of a Sami research ethics committee was to be part of this report.

In April 2009 the white paper Climate for Research (15) was launched.^3^ Here we learn that the SP in consultation^4^ with the government has decided that there is no need for a Nordic panel to report on a Nordic Sami research council. Since Sami research ethics was to be part of this report, this means that that issue is taken off the agenda as well. Instead the SP wants to give priority to a report on how to develop an independent Nordic Sami educational and research institution, including a discussion on how the Sami University College could develop into an indigenous university. A panel (16) appointed in 2010 to report on Sami higher education and research was asked to specifically report on this issue. Even though research ethics was not mentioned in their mandate, the panel briefly discusses the subject in their report and suggested a common Nordic education for researchers in Sami issues to be established.

To ensure that we did not miss any new initiative due to a new executive council of the SP, we sent a letter in November 2013 asking to be updated on their work with Sami research ethics. Their answer was that this was to be on the executive council's agenda in January 2014. The council's decision was that there was to be appointed a panel to address ethical guidelines for the use and administration of Sami biological tissues.^5^

Then in September 2014 the SP invited representatives from universities, colleges and other research institutions to a seminar as their first step in formulating their white paper on higher education and research. However, the only mention of research ethics was in the introductory speech by the politician responsible for research and higher education in the SP's executive committee, where she said it was an important issue.

## Ethical guidelines for Sami research – the issue that disappeared from the SP agenda?

The SP, in asking the Ministry in 1997 to follow up their decision, obviously counted on the national politicians and the national research bodies.

The national ethics bodies, as mentioned, responded positively to the SP's decision of 1997 and took some action such as asking the SP to appoint a Sami consultant to REC V since this body was to have responsibility for all health research applications involving Samis. With the reorganization of the national research ethics system in 2007, this arrangement ended. However, we have not been able to find any discussion as to why it was discontinued.

The Ministry's response came two years later, saying they did not see any need to change the existing research ethics review system (12). Thus, the principal issue in the SP decision – the need for a new research review structure, incorporating a Sami research ethics committee and alternative ethical guidelines that included Sami-sensitive aspects – was actually turned down. We have been unable to find any reactions from the SP to the Ministry's response, but we have noticed that Sami research ethics nevertheless continued to be an issue in parliamentary white papers on research in the years to follow.

Why the SP during this year-long process did not follow up the issue of ethical guidelines for Sami research within their own political and administrative system is a cause for wonder. It might be that they realized that the issue would not yet be approved by the national authorities and put their faith in the discussion of the issue in later research panels, which in fact took place, as we have seen. It might also be that they came to the conclusion that the existing research review structure in Norway consisting of the national ethical guidelines, ethics committees and the international agreements of the Helsinki Declaration (17) were in fact adequate – at least for the time being. Therefore they were satisfied with being entitled to appoint a consultant to the Northern Norway Regional Committee for Medical and Health Research Ethics and being represented on the board of the RCN. Alternatively, they may have viewed the issue in a different light after some years and come to the conclusion that research ethics were primarily an issue for the research community and hence an issue for the proposed Nordic education for researchers to address. It might also be that the overarching discussion of Sami rights in this period in connection with the work on the Finnmark Act, the United Nations Declaration on the Right of Indigenous Peoples and the Nordic Sami Convention had to be prioritized. It is worth noting that Article 27 of the 2005 draft for a Nordic Sami Convention states that research involving Sami interests has to be in accordance with ethical guidelines that correspond with the status Samis have as an indigenous people. Including the issue of Sami research ethics in a Nordic Sami Convention might be interpreted as a deliberate political choice of an alternative agenda to the national ones.

The Sami scholar Harald Gaski has reflected upon the difference in the indigenous discourse on research between the Nordic region and North America and Oceania. The intense debate on methodology and the continuous struggle to “indigenize” academia we have witnessed in North America and Oceania has, to a large degree, been left unfought in the Nordic region. What has been prioritized, he argues, is access to scholarship and institution building (18). Looking at the Sami discourse on research over the last decades, one clearly sees that the main focus has been on the Sami people's right to have their own research institution in order to do research themselves (19–22). Following Gaski, one might say that the SP decision in 2009 to give priority to a panel to report on how to develop an independent Nordic Sami educational and research institution, including a discussion on how the Sami University College could become an indigenous university, is an example of how institution building is favoured.

Making priorities is the task of political bodies, and many times politicians have to be pragmatic as well. The public however expects political bodies to justify their decisions. The SP's justification in 2009 for not having a panel discuss ethics for Sami research was that they did not see it as desirable or expedient. Internationally there are quite a number of examples of guidelines as well as experiences of research review structures involving indigenous peoples’ perspectives (23–26) that could have been used as a frame of reference for the SP.

Whatever the motives for the SP during those years, political pragmatism or political priorities, the outcome is that the research ethics system with regard to Sami research is, with minor adjustments, the same as when the SP asked for a revision of it. No specific ethical guidelines for Sami research have been drawn up and the national guidelines make no specific mention of research involving Samis.

Gaski poses the question of whether this focus on institution building and thereby access to scholarship has been given priority over the content and quality of the scholarship, resulting in the notion of a difference in principle between research politics and research practice. If we follow Gaski in order to understand the SP decision to take research ethics off the agenda, a question that comes to mind is whether the SP today does not see research ethics as connected to research politics, but belonging to the realm of research practice, that is, an issue for the research community only.

## Research ethics – an issue for the research community only?

The ethical obligation of research includes a multiple set of values, norms and institutional arrangements with the purpose to regulate scholarly activity. Firstly, there are norms of the research process itself such as academic freedom. With academic freedom comes responsibility and there are thus also norms that regulate the relationship to those included in the research. A third set of norms are those of relevance or public utility of the research results (27).

In arguing that research could not be seen as separate from the society it operates within, one can say that the SP in 1997 called upon these research ethics norms, especially the responsibility of research to build a relationship with those involved. In their view those involved were not to be understood only as individual Samis, but that research was a concern for the Sami society as a whole.

In spite of years of panels and white papers focusing on Sami research and research ethics, we are left with the situation that the concept of the participation of indigenous communities in research is not an integral part of the Norwegian ethical guidelines. The task of the research ethics boards is still to examine whether the rights of individual research participants are upheld. This is in contrast to developments in, for example, Australia, New Zealand and Canada, where there is an understanding of the protection of Indigenous communities as well as individuals (25). Thus, in Norway it is up to the individual researcher or research institution^6^ to decide whether and how to involve the indigenous community perspective in their research projects.

In our opinion, research ethics is not a topic that should be left to the research community alone to discuss. Research is not an objective activity, it is highly contextual. As researchers we are heavily influenced by the established traditions that prevail in academic institutions. We might therefore not fully comprehend the possible ethical challenges of our research for the individuals or societies we are addressing. We also have to take seriously the fact that research can be associated with colonialism and racism (1, 28) (29) and that the historical power imbalance between the scholarly world and indigenous communities is still in existence.

One of the outcomes of the international indigenous discourse on the role of research was that it was not enough to establish conditions that enabled indigenous people to participate in research themselves. In order to include indigenous peoples’ own knowledge, a transformation of the mentality in the scholarly world was needed. A new paradigm, a new way of understanding had to be introduced (18).

With a new way of understanding come new challenges. When the indigenous world argues that the community perspective be included in guidelines for research ethics, it points to a difference in cultural values (24). Thus, the possible tension between the guiding principles of individual autonomy and collective community rights has to be addressed. Internationally there are experiences of how community sensitive guidelines are practiced to provide for what in the literature are referred to as authentic research relationships (23).

In another article (10), we have discussed our own experience in practicing ethical guidelines that include indigenous community aspects. One of the challenges we as researchers saw in developing a research project in partnership with the Norwegian reindeer herders’ organization on psychosocial distress among reindeer herders was how make sure that their body of knowledge was included in the research process. If we were to succeed we had to establish genuine research collaboration within a framework of mutual trust and cooperation. The ethical guidelines that we turned to were those developed by the Canadian Institute of Health Research (CIHR^7^). The guidelines are intended to assist researchers in upholding indigenous values and traditions, while making them aware of any special considerations that might arise when carrying out research involving indigenous peoples. To us as researchers, the guidelines’ articles with their comments helped us to focus on potential cultural differences in knowledge systems and to understand and accept the scepticism towards research of many in this community. In their opinion, their knowledge of reindeer herding had seldom been seen as knowledge on a par with scholarly knowledge, resulting in research that they saw as biased. The main lesson learned was that involving the indigenous community in research led to a new way of understanding for both parties: the researchers and the community.

When using these guidelines we were fully aware that the indigenous communities in Canada and Norway differ in culture and organization. The legislation and policies that govern the relation between the nation state and the indigenous population in Canada and Norway also differ. In order not to ignore the reality of inter-cultural as well as intra-cultural differences, we would argue that separate research ethics guidelines have to be developed for each specific cultural context.

As long as the SP argues that research is decisive for the development of contemporary Sami society, they should also take on the responsibility of clarifying the values they want to guide scholarship, whether the guiding principle should be that of individual autonomy or of the collective community. Thus, research ethics should not be left to the research community alone to discuss. The new initiatives taken by the SP in 2014 – to set up a panel to address ethical guidelines for the use and administration of Sami biological tissues and to launch a white paper on research that also will address research ethics – tell us that ethical guidelines for Sami research are not totally out of the SP's vocabulary.

The research community on the other hand also has to bear the responsibility to address how to carry out ethical and culturally sound research that involves indigenous communities. The fate of the SP's decision of 1997 should be a reminder that this is not a straightforward task. In line with Glass and Kaufert (26), we would argue that the research ethics review system is heavily influenced by the established institutional frameworks in which it operates, with their shared cultural, methodological and ethical perspectives. Furthermore, we maintain that the indigenous perspective of research fails to be addressed because the main focus in the ethics review system is individual autonomy.

So far the international indigenous and academic discourse on indigenous research ethics does not seem to have had any substantial influence on either the SP or the research communities in Norway. Despite the fact that the research community from time to time has held seminars focusing on Sami research ethics (11, 30–32), research ethics with regard to the Sami population cannot be said to be high on the Norwegian research community's agenda today.^8^ If ethical research guidelines that also include the indigenous perspective as a premise are to be developed, both parties – scholars and Sami politicians alike – have to be strong advocates for this work and be willing to enter into a dialog. Since the SPs in Finland and Sweden are now proceeding with the issue and also want a dialog with the Norwegian SP, there is hope that this dialog will result in an agreement among the three parliaments to pursue the work on ethical guidelines for Sami research.

## Footnotes

1 The Sami Parliament (Sámediggi) is an elected assembly that represents the Sami population in Norway. Thirty-nine members of parliament are elected from seven constituencies every fourth year, at the same time as the national election to the Norwegian Parliament (Stortinget). A separate electoral roll has been established for Sami parliamentary elections. All Sami above the age of 18 are entitled to register in the electoral roll and vote in Sami parliamentary elections. There are also Sami Parliaments in Sweden and Finland.

## Footnotes

2 The Social and Health Committee of the Finnish Sami Parliament stated September 11th, 2014 the importance of ethical questions being clarified and asked their administration to be in dialog with the Norwegian Sami Parliament on the issue. The executive board of the Swedish Sami Parliament noted in their meeting 9 December 2014, the lack of Sami competence in ethical review boards, and decided to proceed with the issue.

## Footnotes

3 Since the last white paper on research, Norway has elected a new government. The Bondevik II government left office in the autumn of 2005 to be replaced by the Stoltenberg I government. In the 2007 elections Stoltenberg II was voted in and remained in office until autumn 2013.

## Footnotes

4 As an indigenous people the Sami have a right to be consulted on issues that may have a direct bearing on them. The Norwegian Government and the SP have signed an agreement specifying how consultation is to take place.

## Footnotes

5 The background for this decision is that the Center for Sami Health Research at the University of Tromsø had called upon the SP to take part in establishing guidelines for the use of the biological tissues they possess, which researchers worldwide have been interested in getting access to. The parliamentary conference of the three Sami parliaments also addressed this issue in 2011, stating their concern about the potential exploitation of indigenous genetic resources (www.sametinget.se/78433).

## Footnotes

6 Sami University College has, as a member of WINHEC, adopted their guidelines for research ethics in indigenous societies (7).

## Footnotes

7 The CIHR guidelines were in effect from May 2007 until December 2010. Health research involving indigenous people in Canada is now governed by the Tri-Council Policy Statement: Ethical Conduct for Research Involving Humans (TCPC).

## Footnotes

8 On the website of the national ethics committees the key words “ethnic group,” “indigenous” or “Sami” are not mentioned under the column “What are you doing research on?” One has to go to their virtual ethics library to find a discussion on these aspects (33, 34).

## References

[1] Kyllingstad JR (2008). Ideene, “Menneskeåndens universalitet.” Institutt for sammenlignende kulturforskning 1917–1940. institusjonen og forskningen [“The universality of the human spirit.” The institute for comparative research in human culture 1917*–*1940. Ideas, institution and research].

[2] Health Research Council of New Zealand Te Ara Tika. Guidelines for Maori research ethics: a framework for researchers and ethics committee members.

[3] National Health and Research Council/Australian Government (2003). Values and ethics. Guidelines for ethical conduct in Aboriginal and Torres Strait Islander health research. http://www.nhmrc.gov.au/_files_nhmrc/publications/attachments/e52.pdf.

[4] National Health and Research Council/Australian Government (2005). Keeping research on track. A guide for Aboriginal and Torres Strait Islander peoples about health research ethics.

[5] Canadian Institutes of Health Research, Natural Sciences and Engineering Research Council of Canada, Social Sciences and Humanities Research Council of Canada Tri-council Policy Statement. http://www.pre.ethics.gc.ca/pdf/eng/tcps2/TCPS_2_FINAL_Web.pdf.

[6] Alaska Federation of Native Guidelines for Research http://www.ankn.uaf.edu/IKS/afnguide.html.

[7] World Indigenous Nations Higher Education Consortium (WINHEC) (2010). Research standards first edition adopted. http://winhec.org/files/WinHEC_Research_Standards_February%202011.pdf.

[8] Sametingets plenum 29

[9] The Norwegian National Research Ethics Committees https://www.etikkom.no/en/.

[10] Møllersen S, Eira-Åhren IM, Tørres G, Stordahl V, Ledman AL (in press). Developing an adequate questionnaire addressing psychosocial distress in a reindeer herding population: some lessons learned. Ethics in indigenous research – past experiences, future challenges.

[11] The National Committee for Research Ethics in the Social Sciences and the Humanities (NESH) (2002). Samisk forskning og forskningsetikk. Seminarrapport [Sami research and research ethics. Seminar Report].

[12] St.meld. nr. 39 (1998–99) Forskning ved et tidsskille [Research at the dawn of a new era]. http://www.regjeringen.no/nb/dep/kd/dok/regpubl/stmeld/19981999/stmeld-nr-39-1999-.html?regj_oss=1&id=192405.

[13] St.meld. nr. 34 (2001–02) Kvalitetsreformen Om høyere samisk utdanning og forskning [The Quality Reform. On Sami higher education and research]. http://www.regjeringen.no/nb/dep/kd/dok/regpubl/stmeld/20012002/stmeld-nr-34-2002-.html?regj_oss=1&id=196279.

[14] St.meld. nr. 20 (2004–05) Vilje til forskning [Commitment to research]. http://www.regjeringen.no/nb/dep/kd/dok/regpubl/stmeld/20042005/stmeld-nr-20-2004-2005-.html?regj_oss=1&id=406791.

[15] St.meld. nr. 30 (2008–09) Klima for forskning [Climate for research]. http://www.regjeringen.no/nb/dep/kd/dok/regpubl/stmeld/2008-2009/stmeld-nr-30-2008-2009-.html?regj_oss=1&id=556563.

[16] Butenschønutvalget (The Butenschøn Panel) (2012). Langs lange spor. Rapport [Along long tracks. report]. http://www.regjeringen.no/upload/KD/Vedlegg/Forskning/rapporter/Langs_lange_spor-.pdf.

[17] WMA Declaration of Helsinki – Ethical principles for medical research involving human subjects http://www.wma.net/en/30publications/10policies/b3/index.html.

[18] Gaski H (2013). Indigenism and cosmopolitanism: a pan-Sami view of the indigenous perspective in Sami culture and research. AlterNative: Int J of Indig Peoples.

[19] Keskitalo AI (1976). Research as an inter-ethnic relation. Acta Borealia.

[20] Larson K (1988). Ethnopolitics and research. Ethics for the non-native researcher. Acta Borealia.

[21] Kuokkanen R, Minde (2008). Sami higher education and research: towards building a vision for future. Indigenous peoples. Self-determination- knowledge-indigeneity.

[22] Stordahl V, Minde (2008). Nation building through knowledge building: the discourse of Sami higher education and research in Norway.

[23] Bull JR (2010). Research with aboriginal peoples: authentic relationships as a precursor to ethical research. J Empir Res Hum Res Ethics.

[24] Flicker S, Worthington CA (2012). Public health research involving aboriginal peoples: research ethics board stakeholders’ reflections on ethics principles and research processes. Can J Public Health.

[25] George AM (2011). Review of procedures for approval of health studies in northern Canada. Int J Circumpolar Health.

[26] Glass KC, Kaufert J (2007). Research ethics and aboriginal community values: can the two be reconciled?. J Empir Res HumRes Ethics.

[27] The National Committee for Research Ethics in the Social Sciences and the Humanities (NESH) (2006). Guidelines for research ethics in the social sciences, law and the humanities. https://www.etikkom.no/Aktuelt/publikasjoner/Guidelines-for-research-ethics-in-the-social-sciences-law-and-the-humanities/.

[28] Åhrén M, Chakma S, Jensen M (2001). Racism and racial discrimination against the indigenous people in Scandinavia and Russia – the Sami people. Racism against indigenous peoples.

[29] Ledman AL (2012). Att utmana koloniala strukturer – etik i samiskrelaterad forskning [Challenging colonial structures – ethics in Sami research]. Bårjås.

[30] Broadbent N, Karlsson H (2004). The ethics of collaborative research in Sweden. Finding common ground with local and indigenous peoples. Swedish archeologists on ethics.

[31] Zachrisson I, Karlsson H (2004). Archeology and ethics. The south Sámi example. Swedish archeologists on ethics.

[32] Ledman AL (in press). Ethics in indigenous research – past experiences, future challenges.

[33] Ingierd H, Fossheim HJ De nasjonale forskningsetiske komiteene [The Norwegian National Research Ethics Committees]. http://www.etikkom.no/FBIB/Temaer/Forskning-pa-bestemte-grupper/Etniske-grupper/.

[34] Niemi E, Semb AJ Forskningsetisk kontekst: historisk urett og forskning som overgrep [The research ethics context: historical injustice and research as abuse]. http://www.etikkom.no/FBIB/Temaer/Forskning-pa-bestemte-grupper/Etniske-grupper/Forskningsetisk-kontekst-Historisk-urett-og-forskning-som-overgrep/.

